# Biodegradable PBAT/PVP Nanofibers with Herbicide Activity against Brachiaria (*Brachiaria decumbens*) Grass Obtained by Solution Blow Spinning

**DOI:** 10.1021/acsomega.6c03394

**Published:** 2026-06-09

**Authors:** Ana C. C. Lemos, Kelvi W. E. Miranda, Júlio C. Ugucioni, Lucas R. F. Figueiredo, Heloisa O. Santos, Joaquim P. Silva, Eliton S. Medeiros, Juliano E. Oliveira

**Affiliations:** † Graduate Program in Biomaterials Engineering (PPGBiomat), Federal University of Lavras, 37200-900 Lavras, Minas Gerais, Brazil; ‡ Laboratory of Research and Innovation in Plant Products and Packaging (LabINOVA), Department of Food Engineering, Federal University of Ceara, 60356-000 Fortaleza, Ceara, Brazil; § Institute of Rural Development (IDR), University for International Integration of the Afro-Brazilian Lusophony, 62790-970 Redenção, Ceara, Brazil; ∥ Department of Physics (DFI, Federal University of Lavras, 37200-900 Lavras, Minas Gerais, Brazil; ⊥ Department of Materials Engineering (DEMAT), Federal University of Paraíba, University City, 58051-900 João Pessoa, Paraíba, Brazil; # Department of Agriculture (DAG), Federal University of Lavras, 37200-900 Lavras, Minas Gerais, Brazil; ∇ Department of Chemical and Materials Engineering (DQM), Federal University of Lavras, 37200-900 Lavras, Minas Gerais, Brazil

## Abstract

Although Agriculture 4.0 combines biotechnology, information technology, and nanotechnology to boost productivity and sustainability in farming, controlled delivery of herbicides is still a challenge. However, nanomaterials are drawing more interest for their potential in developing more precise controlled release systems for agrochemicals. In this work, nanofibrous mats of biodegradable poly­(butylene adipate coterephthalate) (PBAT) and poly­(vinylpyrrolidone) (PVP) blends containing Indaziflam were prepared by Solution Blow Spinning for control release in agriculture. Blends were analyzed by scanning electron microscopy, Fourier-transform infrared spectroscopy, X-ray diffraction, and thermogravimetric analysis. Nanofibrous mats were also analyzed for their biodegradation profile using the aerobic method of Bartha’s respirometer and subjected to the simulation of the potential inhibitor in grass germination. These nanofibers had average diameters ranging from 288 to 367 nm, depending on the concentration of PVP. In this context, nanofibrous mats with varying amounts of PVP exhibited greater biodegradability compared to films. Furthermore, the nanofibrous mats with herbicides showed germicidal action on grass. Therefore, our results indicated that by controlling the amount of PVP in PBAT/PVP blends, controlled release systems of herbicides with potential uses in agriculture can be easily tailored.

## Graphical abstract

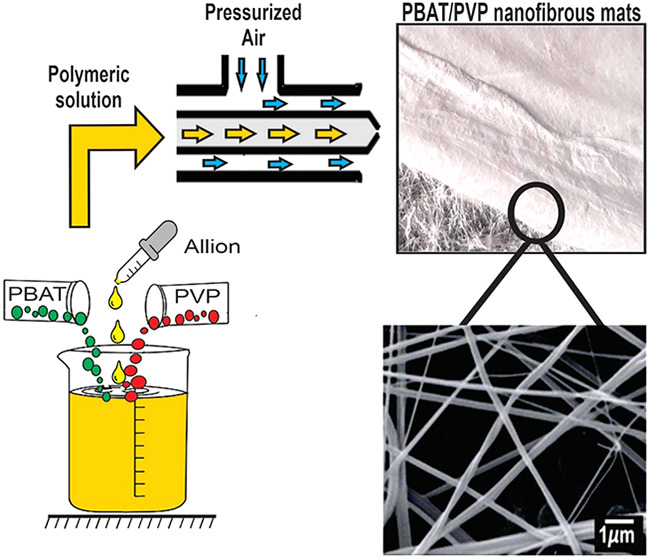

## 1 Introduction

The Food and Agriculture Organization (FAO) projects that the global population will rise from over 7 billion to 8.7 billion by 2033, reaching 9.5 billion by 2050.^1,2^ Urbanization and higher incomes are expected to accelerate, leading to various social issues including challenges in agriculture. In response to these ongoing social challenges and urgent needs, the FAO suggests that food production be increased by at least 70% to effectively address these issues.^3,4^ Moreover, climate change poses significant challenges to food production by directly affecting the supply chain and limiting environmental resources.^3^ Alongside concerns regarding the environmental consequences of indiscriminate herbicide application, these chemicals are also employed to enhance efficiency and safety in agricultural production. However, they do not guarantee the sustainability standards that correspond to SDG 12. Twelve.^5^ In this way, innovation and new technologies appear as supports to solve and optimize such production processes.

New agricultural technologies offer several benefits: (i) improved efficiency with raw materials and greater crop productivity and resistance; (ii) higher product quality and safety with traceability; (iii) better management and decision support; (iv) reduced fatigue and enhanced working conditions; (v) lower environmental impact; and (vi) increased coordination and collaboration among workers in production chains.^6,7^ These technological changes are associated with agriculture 4.0, and the innovative investments are essential for worldwide agribusiness to reduce the distance to technological frontiers.

Environmental impacts caused by traditional polymers have prompted extensive research efforts within laboratories and industry. These efforts focus on identifying polymers and biopolymers derived from renewable sources and utilizing innovative techniques and methods to enhance their properties.^8–10^ To achieve this aim, polyesters have been studied extensively because of their accessibility and low-cost production routes, such as fermentation. These materials offer the advantage of being biodegradable,^11,12^ which makes them suitable for sustainable agricultural applications. The main polyesters used in agriculture are poly­(lactic acid) (PLA), polyhydroxyalkanoates (PHA and PHB), poly­(butylene succinate) (PBS), poly­(caprolactone) (PCL), poly­(ethylene terephthalate) (PET), and poly­(butylene adipate-*co*-terephthalate) (PBAT). Among the copolyesters, PBAT, commercially known as Ecoflex, combines aliphatic and aromatic monomers with its backbone to promote biodegradable and compostable characteristics. Moreover, PBAT stands out for combining sustainability and versatility in blends with a molecular structure that, by delaying crystallization, optimizes the stretching of finer, stronger, and more intact nanofibers during solution blow-spinning.^13^

Another polymer with large potential for blend formation is the poly­(vinylpyrrolidone) (PVP), which is a vinyl polymer with higher hydrophilicity and biocompatibility, widely employed in packaging in the food industry.^14,15^ PVP is an amorphous polymer that forms a stable complex with other polymers and surfactants in the organic solvents. PBAT/PVP blends have received limited attention in the literature; further research is needed to evaluate their environmental impact and potential as substitutes for synthetic polymers in agribusiness.

Polymer processing techniques have been improved to enhance properties and produce materials with controlled structures such as in films, particles, and micro- and nanofibers.^16^ Solution blow spinning (SBS) stands out as an emerging technology to produce from macro- to micro-, submicro-, and nanofibers. This process relies on a parallel concentric fluid flow to form fibers^17,18^ whose characteristics (diameter, shape, smoothness, etc.) depend on both material (polymer molecular weight, solvent flash point, concentration, and viscosity of the polymer solution) and process variables (nozzle geometry, ejection rates, gas pressure, working distance, and collector shape and movement).^19–21^ Compared to electrospinning, SBS offers higher fiber production rates, a greater choice of solvents and targets, along with lower cost and ease of operation.^22–24^ Furthermore, SBS has been explored for agricultural and postharvest applications, including encapsulation of bioactive agents in nanofibers used for crop protection and preservation systems.^25–27^ These studies highlight the versatility of the SBS technique and its potential for developing innovative delivery platforms for agrochemicals.

Biodegradable polymer nanofibers represent a promising platform for the encapsulation of synthetic herbicides, with the aim of improving weed management. Solution blow-spun nanofibers provide high surface area/volume ratios and adjustable porosity, allowing controlled and environmentally friendly release, responsiveness to stimuli, protection of active ingredients against photolysis and microbial degradation, and increased root absorption. Encapsulation in biodegradable polymeric matrices, such as PBAT, a relatively hydrophobic polyester, can limit water diffusion and influence the release behavior of encapsulated compounds, reducing the mobility and exposure of nontarget organisms, decreasing the number of required doses, and mitigating the risks associated with herbicide application toward safer and more sustainable agricultural practices to safeguard human health.^25,27^ In addition to maintaining herbicide efficacy, this approach integrates strategies to modulate the release and adjust release kinetics to fit agronomic needs in a sustainable and precise manner.

Biodegradation involves breaking down polymer chains, resulting in a decrease in the molecular weight. These chains can be reduced to monomers, which are then consumed by bacteria or fungi in water.^28^ The process is aided by the low bond energy of the components.^29^ Respirometry, which measures CO_2_ generated during degradation, is a common method for assessing polymer biodegradability. As polymers biodegrade, encapsulated or aggregated components may be gradually released. Biodegradable nanofibrous mats in agriculture can deliver micronutrients or herbicides slowly, reducing chemical runoff and preventing soil contamination.^30^ Although the literature presents exploratory studies of nanofiber-based delivery systems, the use of biodegradable polymer blends produced by SBS for the controlled release of herbicides and the impact on biodegradability are still limited, especially regarding the investigation of the relationship between polymer composition, fiber morphology, and biodegradation behavior in nanofibrous systems designed for agricultural applications.

The aim of this work was to obtain PBAT/PVP nanofibrous blend mats with herbicide activity obtained by solution blow spinning. The investigations of nanofiber mats and the biodegradation profile were compared to cast films. The biodegradation tests were performed by an aerobic method using the Bartha respirometer and were used as a simulation of chemical delivery.

## 2 Experimental Section

### 2.1 Materials

Poly­(butylene adipate coterephthalate), PBAT, (Ecoflex F Blend C1200, *M* = 6.6 × 10^5^ g/mol) was purchased from OEKO (SC, Brazil). Poly­(vinylpyrrolidone) (*M* = 360,000 g/mol, CAS 9003-39-8), PVP-K360, was obtained from Sigma-Aldrich (USA). Chloroform P.A.-ACS (CAS 67-66-3) was purchased from LabSynth (SP, Brazil). Potassium hydroxide lentil P.A.-ACS (*M* = 56.11 g/mol, CAS 1310-58-3), hydrochloric acid 37% P.A.-ACS (CAS 7647-01-0), and barium chloride dihydrate P.A.-ACS (*M* = 244.26 g/mol, CAS 10326-27-9) were obtained from Exodo Scientific (SP, Brazil). Phenolphthalein P.A. (*M* = 318.32, CAS 77-09-3) was purchased from Neon (SP, Brazil). Indaziflam was obtained from Bayer (RJ, Brazil). *Brachiaria decumbens* seeds cv. Basilisk were acquired from Matsuda (SP, Brazil).

### 2.2 Films and Nanofibers

Nanofibrous mats and films of PBAT, PVP, and PBAT/PVP blends were obtained by using an experimental design described in Table 1. The precursor solutions of PBAT/PVP were prepared in a total concentration of 10% w/v in chloroform (1 g of polymer in 10 mL of chloroform). PVP was added to chloroform under stirring until it was completely dissolved. This order of addition is a classic strategy in the dissolution kinetics and thermodynamics of mixtures and is crucial when dealing with polymers with such distinct behaviors as PBAT and PVP. Then, PBAT was added, and stirring was maintained for 1 h at 500 rpm at room temperature in hermetically sealed flasks to control solvent evaporation. The resulting solution is macroscopically transparent, but at the molecular level, it may be partially transparent. The casting methodology was also adopted for the film preparation.^31^ These solutions were poured into Petri dishes in a hood for 36 h at room temperature until complete solvent evaporation and film formation occurred.

**Table 1:** Experimental Design of the Precursor Solutions of PBAT, PVP, and PBAT/PVP Blends

sample	PBAT (%)	PBAT (g)	PVP (%)	PVP (g)	PBAT:PVP ratio
PBAT/PVP-0	100	1	0	0	1:0
PBAT/PVP-25	75	0.750	25	0.250	3:1
PBAT/PVP-50	50	0.500	50	0.500	1:1
PBAT/PVP-75	25	0.250	75	0.750	1:3
PBAT/PVP-100	0	0	100	1	0:1

Nanofibrous mats were obtained by the Solution Blow Spinning technique (SBS)^17^ with some modifications in the experimental apparatus (Figure 1). Polymer and blend solutions were injected by an injection pump (New Era Pump Systems, NE-300 model) at 6 mL/h using a glass syringe with an organic solvent-resistant capillary (FLURAN F-5500-A, Ismatec, Wertheim, GER) and a metal needle with inner and outer nozzle diameters of 0.5 and 1.2 mm, respectively, and a protrusion length of 2 mm. An air compressor (Chiaperini, 000784 12BPV 160 L, 1HP-4P, oil-free) was used to provide a constant pressure of 414 kPa for nanofiber production. The spun mats were collected using a rotating collector wrapped with aluminum foil and positioned 20 cm from the nozzle outlet. The SBS apparatus was placed in a box to control the temperature at 37.5 ± 0.5 °C and the relative humidity (RH) at 70 ± 2%. The SBS spinning parameters were kept constant for all samples.

**Figure 1:**
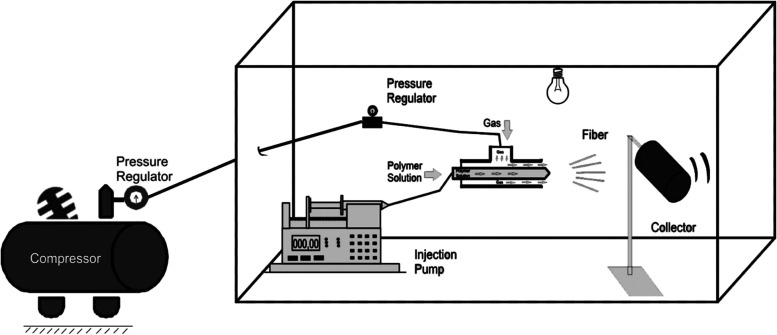
Solution Blow Spinning (SBS) experimental apparatus used to prepare polymer/blend fibrous mats.

### 2.3 Characterization

The morphology of the SBS fibers was analyzed using scanning electron microscopy (SEM) model EVO 40XVP (Leo Electron Microscopy Ltd., Carl Zeiss, Cambridge, UK). Samples were mounted on aluminum stubs and sputtered with gold (Bruker and cryosystem Gatan, Alto 1000, Massachusetts, EUA). Average fiber diameters were measured using ImageJ (National Institutes of Health, Bethesda, MD) from 100 random measurements.

**Figure 2:**
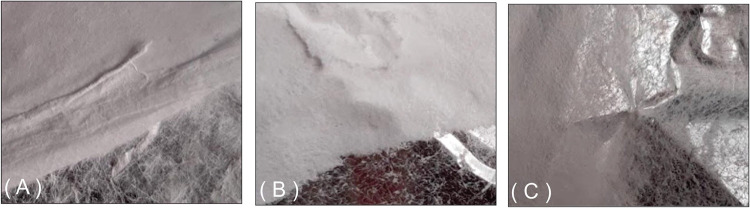
Visual aspect of the nanofibrous mats collected over aluminum foil: (A) PBAT/PVP-0, (B) PBAT/PVP-25, (C) PBAT/PVP-50. The PBAT/PVP-75 and PBAT/PVP-100 samples did not form nanofibrous mats with the same parameters as the other samples. Images have a horizontal size of about 3 cm.

Fourier Transform Infrared spectroscopy (FTIR) was performed using a Shimadzu spectrometer (IR Affinity-1 FTIR8400S, Kyoto, Japan). Spectra were recorded on the attenuated total reflectance (ATR) mode, 64 scans and a spectral resolution of 4 cm^–1^ in the range of 500–4000 cm^–1^.

X-ray diffraction (XRD) analysis was performed to investigate the crystallinity degree of films and nanofibrous mats. X-ray scans were carried out from 5 to 60° (2θ) at a scan rate of 2 deg/min using Ni-filtered Cu Kα radiation (wavelength at λ = 0.154 nm) at 40 kV and 30 mA. The patterns were recorded using a Rigaku (Miniflex II) X-ray diffractometer. The full-width at half-maximum height (fwhm) of the diffraction peaks was calculated by fitting the X-ray diffraction patterns with a Lorentz–Gaussian function using the Origin 7.5 software (Origin Lab, USA).

Thermogravimetric (TGA) and derived thermogravimetric (DTG) analysis were performed on a Shimadzu (DTA-TG 60 H) analyzer under a nitrogen atmosphere at 50 mL/min, subjected to an ambient temperature range to 700 °C, and a heating rate of 10 °C/min.

### 2.4 Biodegradation

Samples were submitted to a respirometry test of carbon dioxide (CO_2_) quantification of microbial activity during the biodegradation process, according to Brazilian Standards (NBR 14283–ABNT).^32^ This test was previously proposed by Bartha and Pramer.^33^ Two 2 cm^2^ samples were put in the biometer flasks containing 50 g of soil, which were incubated at 28 ± 2 °C for 120 days. CO_2_ was measured volumetrically in a range of 24 h, by titration with hydrochloric acid. The amount of CO_2_ in respirometry was calculated by eq 1:

1 $$\mathrm{mg}\;\mathrm{of}\;{\mathrm{CO}}_{2}=\left(A-B\right)\times 50\times 0.044\times f_{\mathrm{HCl}}$$

where *A* is the solution volume of consumed HCl in KOH for blank (mL); *B* is the solution volume of consumed HCl in KOH for respirometry with polymer (mL); *f*_HCl_ is the factor of solution of HCl. The parameters 50 and 0.044 are related to transformation factor to μmol and mg for CO_2_, respectively.

### 2.5 Germination Test Simulation

*Brachiaria* grass (*B. decumbens*) is the most prevalent weed as it competes with crops for essential nutrients, leading to decreased yields. This species exhibits rapid growth and significant proliferative capacity, which necessitates effective management and control strategies. Among them are herbicides such as indaziflam, which is the active substance present in Alion, used to control pre-emergent weeds in different crops.^34^ Indaziflam acts directly in controlling grasslike *Brachiaria* and other species of grasses. However, its persistence in the soil poses a high risk of residual effects (carryover) that may impact subsequent crops.^35^ Slow-release systems reduce herbicide handling, lower usage, and minimize evaporation and groundwater contamination, making the product more eco-friendly and economically valuable. Slow-release systems reduce herbicide handling, lower usage, and minimize evaporation and groundwater contamination, making the product a more eco-friendly and economically valuable product.

Germination tests were performed on grasses of *B. decumbens* seeds. Nanofibrous mats were prepared with 0.02 g/mL (suggested dose by the Brazilian Ministry of Agriculture, Livestock and Supply) of the herbicide (Indaziflam) through the experimental procedure described in section 2.2. Thus, 0.2 g of herbicide was used for a 10% w/v solution (1 g of polymer to 10 mL of chloroform). The herbicide was added during the solution homogenization process and spread on absorbent paper, where germination tests were performed. The germination test was performed with 8 repetitions of 50 seeds. Sowing was carried out on two sheets of blotting paper in Gerbox boxes; the papers were moistened with a volume of water equivalent to 2.5 times the dry weight of the paper. They remained in BOD chambers with an alternating temperature of 20–35 °C and a photoperiod of 8–16 h.^36^ Readings were taken at 7 and 14 days after showing. The results were expressed as a percentage of germinated seeds.

The treatments consisted of a positive control, where the concentration of the herbicide was diluted in water to wet the papers, three treatments with PBAT/PVP in solutions of 0, 25, and 50 wt %. The 0 wt % concentration was considered as the negative control.

### 2.6 Statistical Analysis

Data was statistically analyzed using Statistica Software Version 12 (StatSoft. Inc., USA, 2011) using one-way analysis of variance (ANOVA), followed by Tukey’s method at a 5% significance level.

## 3 Results and Discussion

### 3.1 Characterization of SBS Nanofibrous Mats

The PBAT nanofibrous mats without and with different PVP concentrations (25 and 50 wt %), obtained by the SBS technique, are shown in Figure 2. However, samples with a high concentration of PVP (PBAT/PVP-75 and PBAT/PVP-100), obtained with the same parameters as those for PBAT/PVP-25 and PBAT/PVP-50 blends, did not promote nanofiber formation.

Figure 3 shows the SEM micrographs of PBAT and PBAT/PVP nanofibrous mats (25% and 50 wt % of the PVP concentration) and the average diameter for the samples. Only films were observed at high PVP concentrations (PBAT/PVP-75 and PBAT/PVP-100), as previously noted, and PBAT/PVP-25 exhibited the thickest fibers. This effect may be related to the increased entanglement of polymer chains resulting from the incorporation of PVP into the blend.^24,37–39^ On the other hand, the PBAT/PVP-50 nanofibrous mats showed a decrease in average diameter compared to PBAT/PVP-25 (*p* < 0.05), with small particles in the submicrometer range collected on the surface of the aluminum foil (Figure 3C).

**Figure 3:**
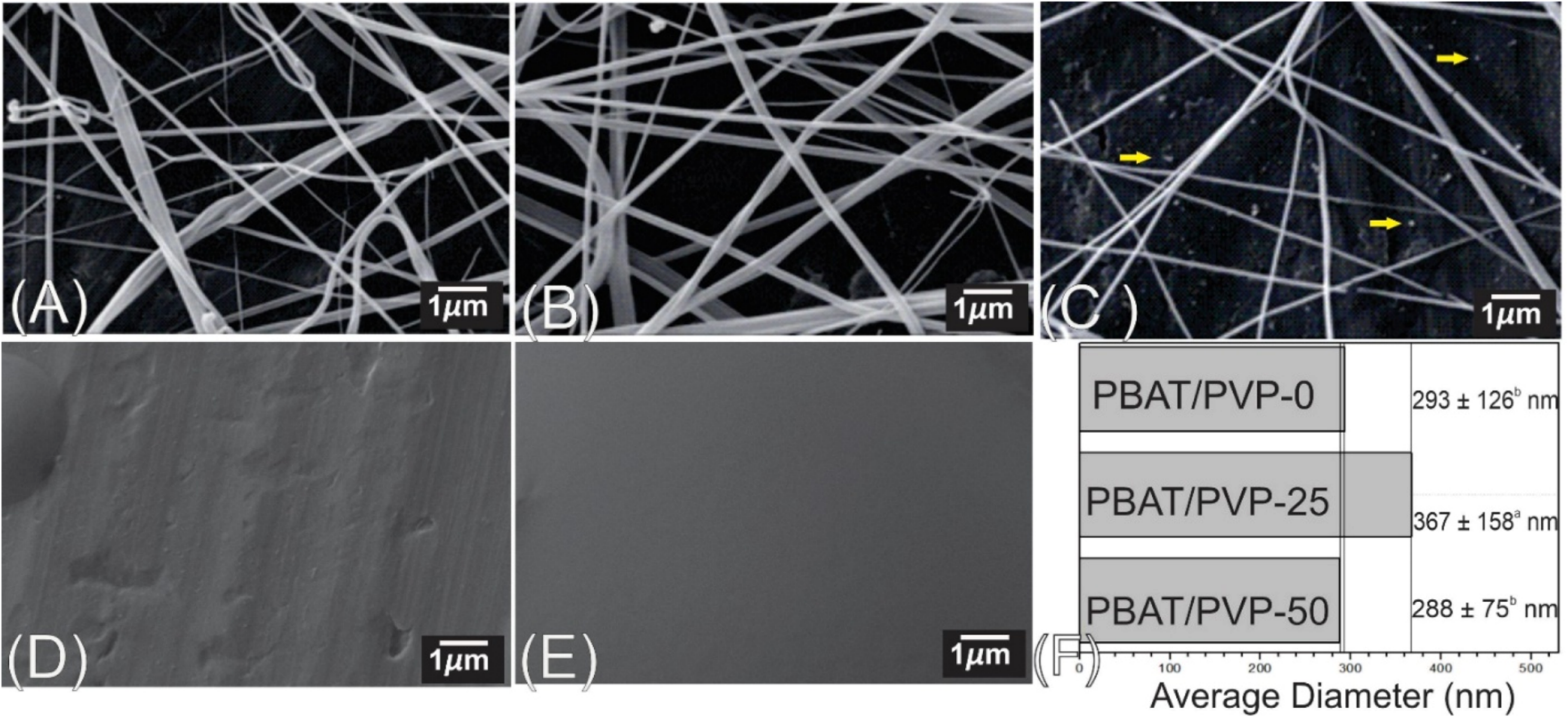
SEM micrographs of the samples: (A) PBAT/PVP-0, (B) PBAT/PVP-25, (C) PBAT/PVP-50, (D) PBAT/PVP-75, (E) PBAT/PVP-100. The average diameter of the nanofibers (F) was obtained using ImageJ software measuring 100 nanofibers. All images were obtained at a magnification of 20,000× with a scale bar of 1 μm.

The difference in average diameter and the formation of submicrometer particles can be attributed to the SBS technique parameters that favor PBAT over PVP whose hydrophilic nature is higher and that can make solvent evaporation more difficult.^24^ However, the measured average diameter did not show a significant difference (*p* ≥ 0.05) with the incorporation of PVP for the PBAT/PVP-0 and PBAT/PVP-50 samples. Other authors have also observed the same behavior.^24^

Analysis of the possible chemical interactions between the polymers PVP and PBAT polymer chains was carried out by FTIR.^24^ PVP is a polymer formed by multiple chains of vinylpyrrolidone, which is a highly hygroscopic and polar polymer,^40,41^ as can be seen in Figure 4A. On the other hand, PBAT is synthesized from 1,4-butadiol, adipic acid, and terephthalic acid polymers, resulting in a copolymer with a molecular structure,^13^ shown in Figure 4B, that has an opposite behavior.

**Figure 4:**
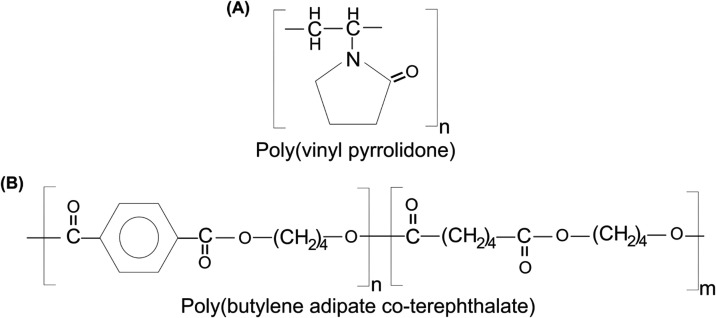
Molecular structure of polymers: (a) polyvinylpyrrolidone (PVP) and (b) poly­(butylene adipate coterephthalate) (PBAT).

Figure 5 shows the FTIR spectra of nanofibers and films compared with the pure polymers. Moreover, films and nanofibers spectra showed differences with each other due to casting and SBS techniques.^42^ Two characteristic bands related to PBAT (red line) can be observed: (i) 1735 cm^–1^ related to ester group ν­(C=O),^43^ and (ii) 1640 cm^–1^ related to ν (phenylene).^44^ Nanofiber PBAT/PVP-0 spectrum (Figure 5A) showed a distinctive band in 1722 cm^–1^, attributed to C=O and phenylene groups. This could be related to the technique used for nanofiber production since polymer chains are rapidly stretched during spinning across the working distance, before reaching the collector as the solvent rapidly evaporates. The wavenumber shifts, suggesting that the phenylene group might become hindered because of PBAT chains, which prevent it from vibrating. Consequently, while the phenylene group appears in the nanofiber, its signals are overlapped on this band. PBAT/PVP-0 spectrum of films (Figure 5B) presented the same bands as for PBAT, indicating that the casting technique did not affect the chemical structure of polymer chains as expected.

**Figure 5:**
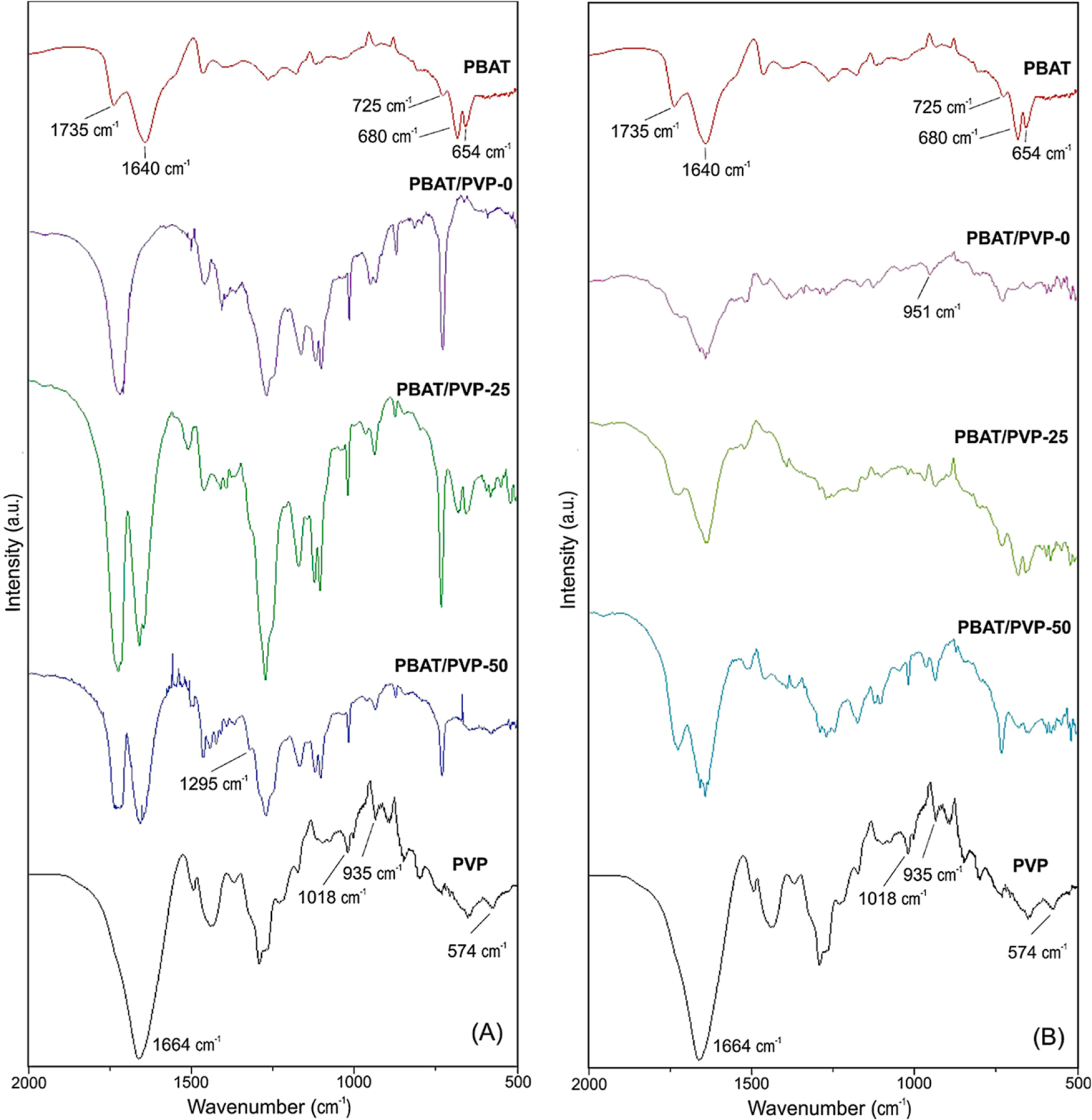
FTIR spectra of PBAT and PVP compared with nanofibrous mats (A) and films (B).

Although the bands between 725 and 654 cm^–1^ were observed for PBAT, the bands at 680 and 654 cm^–1^ are not present in the spectra of nanofibers and films of PBAT/PVP-0. These bands were attributed to the CH_2_ group (725 cm^–1^) and benzene (680 and 654 cm^–1^). The band at 725 cm^–1^ was more evident for nanofibers than for films, which suggests again that the phenylene group was immobilized due to the fast nanofiber production by SBS. Furthermore, the 680 and 654 cm^–1^ bands continued to be observed in films, indicating that phenylene groups have a higher degree of freedom to vibrate.

An indication of the polymer chain stretching direction of the PBAT was observed for bands at 1272, 1165, 1120, and 1105 cm^–1^, which were related to the C–O–C bonding and were more evident in nanofibers than in films. This bond is found in the PBAT backbone (as can be seen in Figure 4B). This result suggests that polymer chains were again oriented during fiber formation by the SBS. On the other hand, casting did not promote chain stretching, so these bands were observed at lower intensity.

With the addition of PVP, the bands 1664, 935, and 574 cm^–1^ related to C=O; C–C bond of CH_2_ and bending of the N–C=O group, respectively, were observed for nanofibers and films. Furthermore, the PBAT/PVP-25 nanofiber presented bands around 1721 and 1657 cm^–1^, where the first is attributed to C=O of PBAT and the second to phenylene of PBAT and C=O of PVP. The apparent increase in intensity of the 1657 cm^–1^ band was associated with PVP addition, and, for PBAT/PVP-50 nanofiber, the same bands are observed, but the band related to C=O of PBAT presented lower apparent intensity in relation to phenylene and C=O of the PVP. Additionally, the spectra for the nanofiber showed bands for PBAT and PVP, indicating that the two components were present in fiber formation. The PBAT/PVP-25 and PBAT/PVP-50 films presented characteristic bands of both polymers.

Therefore, nanofibers present the main characteristic bands of both polymers, proving the blend formation and evidencing complementary bands, which were not found in PBAT and PVP spectra. This can be related to different chain conformations caused by the spinning process, as compared to cast films.

XRD analyses were performed to determine the crystal structure of the samples compared with pure PBAT and PVP. Figure 6 shows that spun samples have a lower degree of crystallinity in relation to films and unprocessed polymer (pellets), as can be seen in Table 2, obtained by deconvolution methods.

**Figure 6:**
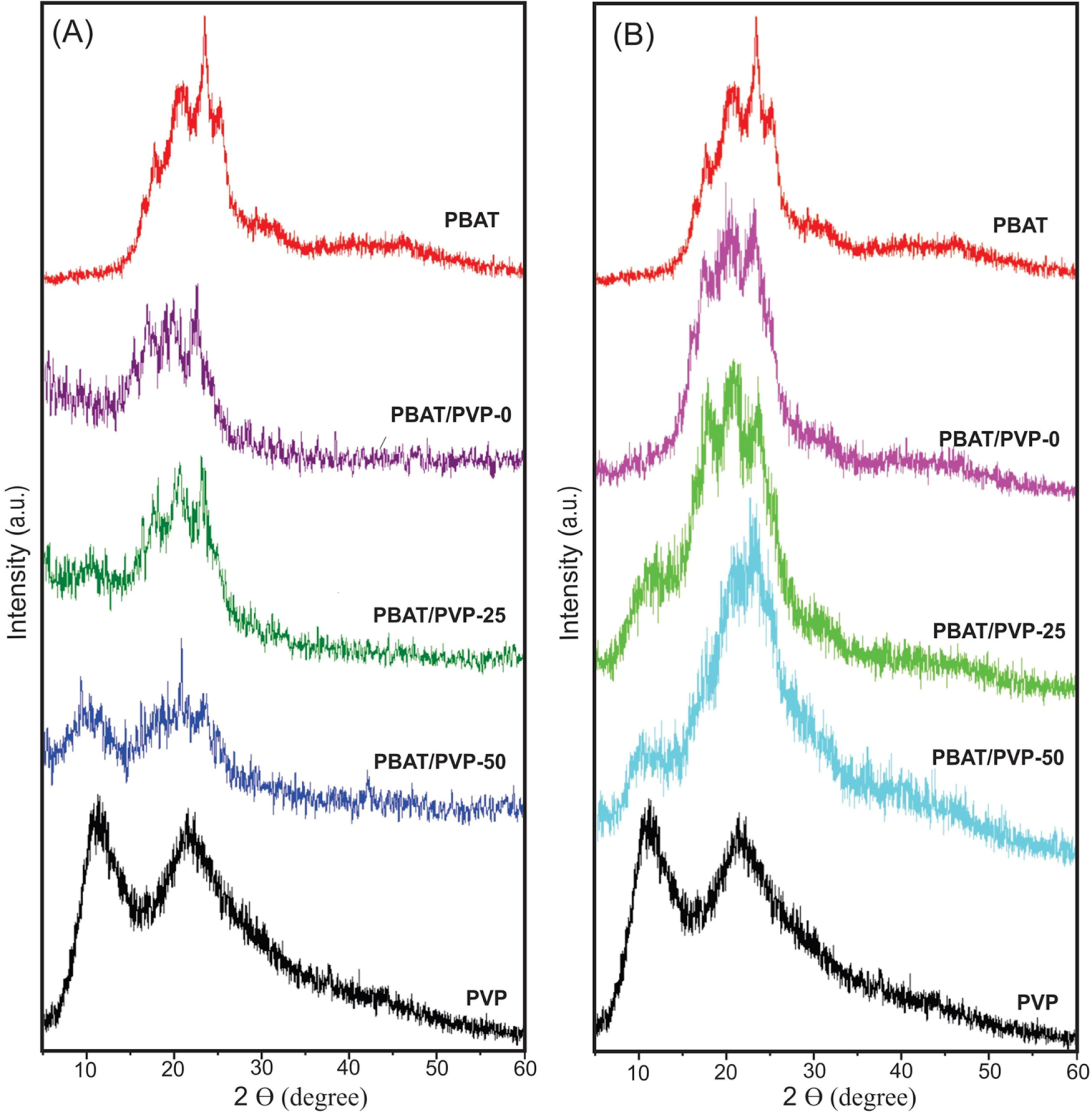
XRD patterns for the polymers and blends: (A) nanofibrous mats and (B) film.

**Table 2:** Degree of Crystallinity in Pure Polymers, Nanofibers, and Films

sample	crystallinity (%)
**Pure Polymers**	
PBAT	40.6
PVP	28.2
**Film**	
PBAT/PVP-0	15.7
PBAT/PVP-25	6.5
PBAT/PVP-50	4.0
**Nanofibers**	
PBAT/PVP-0	1.8
PBAT/PVP-25	14.5
PBAT/PVP-50	0.4

The characteristic inflections of PBAT were observed around 17.6, 20.7, 23.4, and 25.2° ^45–47^ and, for PVP, around 11.2 and 21.5°,^48^ as can be seen in Figure 6. All samples have peaks of PBAT, which decrease with the increase of PVP concentration. Table 2 presents the calculated values of the degree of crystallinity, which is lower for films. An exception is observed for the PBAT/PVP-25 nanofiber, which could influence the results of biodegradation.

A hypothesis of a lower crystallinity degree of nanofibers could be explained as the faster solvent evaporation provided by SBS, which promotes an accelerated solidification of the polymers, hindering the rearrangements of chains, and avoiding the crystallization process.^49^ Conversely, the time of solvent evaporation in the casting process is long enough to promote adequate rearranging of chains to increase crystallinity.^50–52^

Zong et al.^52^ reported similar findings for nanofibrous mats of poly­(lactic acid) (PLA), a semicrystalline polymer. They attributed the retarded crystallization process to the rapid solidification of highly stretched polymer chains under high elongation rates, which hindered crystal formation.^52^ Likewise, Costa et al.^50^ produced nano- and submicrometric fibers of poly­(vinylpyrrolidone) (PVP) and poly­(vinyl chloride) (PVC) incorporating TiO_2_ and ZnO via SBS. According to them,^53,54^ polymer chains are stretched as the solvent rapidly evaporates, resulting in a less crystalline structure. Thus, the SBS technique can effectively modulate the crystallinity of the material, which in turn can be used to tailor delivery systems since a more crystalline structure makes release slower, while a less crystalline one facilitates the controlled release.

Thermogravimetric analyses were used to investigate the thermal degradability of the films and nanofibers and to obtain the decomposition temperature.^55^ Weight loss (TG) and first derivative (DTG) of PBAT, PVP, films, and nanofibers are shown in Figure 7, and temperature values are given in Table 3.

**Figure 7:**
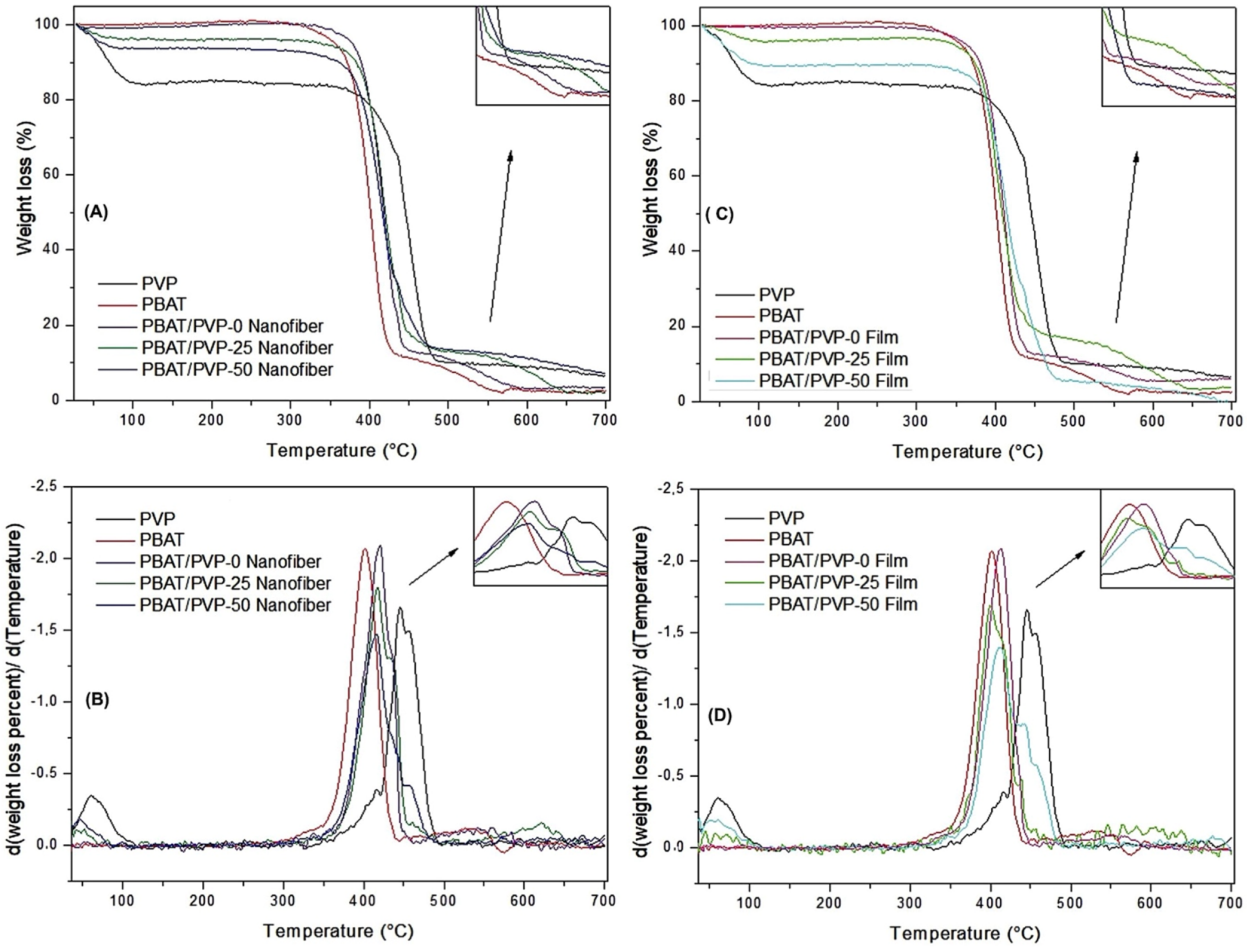
TGA/DTG curves for PBAT/PVP nanofibers (A and B) and films (C and D), respectively.

**Table 3:** Temperature of Decomposition of the Samples: PBAT, PVP, Films, and Nanofibers

	first decomposition		second decomposition			third decomposition		
sample	*T*_onset_ (°C)	weight loss (%)	*T*_onset_ (°C)	*T*_endset_ (°C)	weight loss (%)	*T*_onset_ (°C)	weight loss (%)	residue (600 °C) (%)
**Pure Polymers**								
PBAT pressed			379	419	89.0	536	7.0	2.3
PVP powder	60	14.0	409	457	74.0			8.4
**Polymer Films**								
PBAT/PVP-0			380	420	87.0	511	6.5	5.4
PBAT/PVP-25	60–80	3.5	380	422	80.0	540	12.5	3.5
PBAT/PVP-50	60–80	10.1	380	434	85.0	531	2.0	3.4
**Polymer Nanofibers**								
PBAT/PVP-0			380	419	87.0	513	8.9	3.0
PBAT/PVP-25	60–80	3.5	380	421	83.0	571	10.4	2.1
PBAT/PVP-50	60–80	5.9	380	427	80.0			7.0

PBAT (Figure 7) has a maximum temperature of thermal degradation around 379 °C with a weight loss of 89%. The sequence of the degradation occurred around 536 °C with a weight loss of 7%. These thermal degradation processes were related to the break of the main polymer chain and end groups. The polyesters have several degradable bonds such as carbon–oxygen bond, carbon–carbon bond, among others,^56–58^ and PBAT had two steps of degradation: (i) from 340 to 400 °C related to aliphatic copolyester decomposition (adipic acid and 1,4-butanediol) and (ii) from 520 to 600 °C, terephthalic acid decomposition (aromatic).^59^

PVP also has two degradation steps. The first step around 59 °C is attributed to humidity loss^60^ (weight loss of 14%), and the second step, around 409 °C, is related to the thermal degradation of organic constituents of the polymer (weight loss of 74%).^61^ The degradation processes of the second step takes place by breaking of nitrogen–carbon bonds of the N–C=O groups, following by ammonia release and protonation of nitrogen; in other words, decomposition of the carbon of the pyrrolidine group and main chain degradation.^62–65^

PBAT/PVP blends showed mass loss characteristic of both polymeric matrices, mainly in the first stage, referring to PVP (60–80 °C, Table 3). Moreover, DTG curves (Figure 7B) show a two-step degradation with the presence of two additional peaks attributed to PBAT (1st step: 379–435 °C; second step: from 510 °C, Table 3), indicating that there were no bonds between these polymers as already confirmed by FTIR results.^66–68^ PVP exhibits greater thermal stability compared to PBAT; however, adding PVP to the samples does not alter or enhance the initial degradation step. This observation suggests that separate degradation mechanisms take place in each polymer, and PVP may be dispersed within the PBAT matrix. According to existing literature, binary mixtures can display distinct morphologies.^50,69^

### 3.2 Biodegradation Assay

The results of the biodegradability behavior of the samples are shown in Figure 8, which consists of CO_2_ emission of the respirometry in the inoculum (soil) in order to study the microbial activity/degradability of samples that exhibited a similar overall biodegradation profile over time, characterized by a gradual increase in cumulative CO_2_ production.^30^

**Figure 8:**
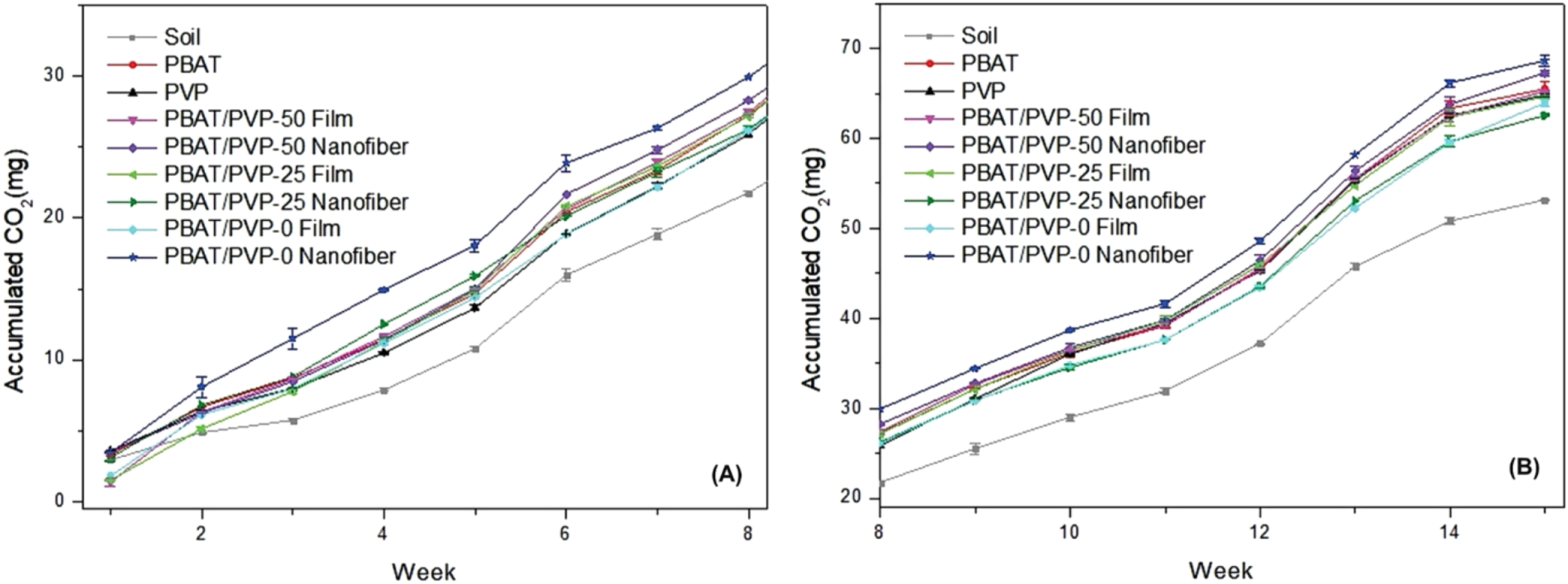
CO_2_ released during the biodegradation process for all samples. (A) 0 to 8 weeks and (B) 8 to 15 weeks.

Figure 8 shows that though curve slopes are similar, final CO_2_ values vary slightly among samples regardless of the PBAT/PVP ratio. Significant differences (*P* < 0.05) in polymer matrices—PVP and PBAT—were seen between weeks 2 and 10, likely because PBAT’s short aliphatic chain structure (adipic acid and 1,4-butanediol) degrades faster than PVP. After week 10, accumulated CO_2_ was similar across all samples, aligning with crystallinity results in Table 2. After the tenth week, there were no differences in accumulated CO_2_ between samples. This is related to the degree of crystallinity results observed in Table 2. Literature shows that biodegradation processes occur due to scission of the main chain into crystalline and semicrystalline domains in polymers.^70,71^

The PBAT/PVP-0 nanofiber mats exhibited a higher cumulative release of CO_2_ during the assay, as indicated by a greater amount of accumulated CO_2_ (Figure 8). However, there were no differences between PBAT/PVP-25 and PBAT/PVP-50 until the fifth week. From the sixth week onward, PBAT/PVP-50 accumulated more CO_2_ than PBAT/PVP-25. These variations may be associated with differences in polymer composition and fiber structure, which can affect the accessibility of biodegradable domains. For example, there is a difference in average fiber diameter (Figure 2F) and degree of crystallinity: PBAT/PVP-25 has a larger diameter and a higher degree of crystallinity than does PBAT/PVP-50. This resulted in a higher packing of nanofibers in the mats, preventing microbial activities.^72–76^

Film biodegradation time was longer than that of the nanofibers due to its lower surface area. Also, the addition of PVP increased microbial activity as compared to samples without this polymer (PBAT/PVP-0). Greater degradability was only seen after 5 weeks, likely due to lower crystallinity in the mixtures. Amorphous regions degrade faster than crystalline domains.^29,71,77,78^ Notably, samples containing PVP exhibited a lower degree of crystallinity than that of the PBAT/PVP-0 samples. Furthermore, PVP is more hydrophilic, which facilitates water absorption and microbial interactions. This slightly increases the total amount of CO_2_ released without significantly altering the degradation kinetics.

The accumulated amount of CO_2_ of all nanofibers was greater than that for films and unblended polymers. The PBAT/PVP-0 nanofibrous mats presented higher degradation in relation to PBAT and the PBAT/PVP-0 film. The morphologic distinction of nanofiber (crystallinity degree of 1.8% and average diameter of 293 nm), film (crystallinity degree of 15.7%), and PBAT pure (crystallinity degree of 40.6%) is related to this effect. Nanostructured materials have a higher surface area, which facilitates the microbial activity, as observed for nanofibers and films.^79^ Therefore, nanofibrous mats are promising alternatives for encapsulating active substances and enabling a controlled release during mat degradation.

In general, nanofibers transport a very small number of active substances when they are used as controlled-release systems. However, because indaziflam is used in very low doses, the SBS system can carry enough herbicide to protect the area without needing a large quantity. In conventional spraying, many products are lost through drift (wind) or leaching. For agricultural commodities and bulk inputs (fertilizers), this technology is still in development; however, for precision agriculture, high-value seed protection, reforestation, and next-generation herbicides (such as Alion), the quantitative limitation is not a mistake but a characteristic. The goal is not to apply more herbicide but to ensure that the small amount applied lasts during the entire crop season.

### 3.3 Application of PBAT/PVP Nanofibrous Mats in Germination Tests

The germination tests of grass (*B. decumbens*) for control (only the paper) and PBAT/PVP-50 nanofiber without and with 0.02 g/mL indaziflam are shown in Table 4.

**Table 4:** Germination Tests of *Brachiaria* Seeds, Using PBAT Nanofibers with Different Concentrations of PVP and 0.02 g/mL of Herbicide (Indaziflam)

	% brachial germination	
sample	7 days	14 days
PBAT/PVP-0	52.8 ± 4.1^a^	93.0 ± 5.0^a^
PBAT/PVP-25	21.0 ± 5.3^b^	48.8 ± 5.0^b^
PBAT/PVP-50	3.3 ± 1.5^c^	5.8 ± 2.5^c^
Control (+)^a^	0^d^	0^d^

a Positive control (0.02 g/mL concentration of herbicide in water). Values represent mean ± standard deviation (*n* = 50). Values that share the same superscript are not statistically different from one another.

Table 4 shows that the nanofibrous mats with herbicides showed greater efficiency in inhibitory action on the germination of *Brachiaria*. However, PBAT/PVP-0 did not inhibit the germination of the seeds. This may be associated with the ability to encapsulate the herbicide by PBAT during the spinning process. According to the literature, encapsulating agents can present different physical-chemical features such as hydrophobicity or hydrophilicity, low hygroscopicity, and good capacity for incorporating materials.^80–82^ Therefore, such observations corroborate the hypothesis that PBAT acts as an encapsulation agent.

The mats with PVP showed great efficiency in the inhibition during the germination period of the seeds with significant differences (*p* < 0.05) (Table 4). Such behavior may be associated with the affinity of PVP with herbicides since both have a hydrophilic profile. This affinity is enhanced by increasing the concentration of PVP in the nanofiber mats, maintaining the behavior of the encapsulated material (herbicide) with the PBAT/PVP nanofibers. However, it is possible that the germicidal action of nanofibrous mats is due to the presence of residues of herbicides on the surfaces of the fibers. During the process of fiber formation and solvent evaporation, some sprinkling of the herbicide on the fiber surface may occur. Thus, it shows a quick response to the germicidal action of the material, during the analysis period of 14 days, by leaching the nanofiber surface.

It is possible that during the process of degradation of the nanofibrous mats, the encapsulated material is gradually released into the environment.^83^ However, the speed of dissemination of herbicides in the environment must be in accordance with the speed of growth of the crop.^84^ That is, fast-growing crops will need nanofiber materials with a higher concentration of PVP, but in the case of slower crops, lower concentrations of PVP, even with the action leaching process (watering period) (Figure 8 and Table 4), the highest concentration of the active material can be found in PBAT nanofibrous mats. Encapsulated herbicides are known to be efficient for a prolonged period without having to use overdoses in the environment.^83^ According to Morota et al.,^35^ the use of encapsulated herbicides during the production of flute trees can reduce weed growth. Faber et al.^85^ demonstrated that the efficiency of indaziflam released into the environment was gradually observed after six months. Gray et al.^86^ showed the effectiveness of indaziflam over four years, and a reduction in quantity and applications was also observed. Ravindran et al.^83^ indicated that controlled-release fertilizers are considered viable in order to mitigate adverse environmental effects of the applied products, as well as to increase the efficiency of nutrient use by the environment.

Although the results suggest that the herbicide was incorporated into the nanofibrous mats, encapsulation was inferred indirectly, based on both the inhibition of germination and the physicochemical affinity between the polymers and the active compound.^87,88^ Future investigations using morphological or spectroscopic mapping techniques, such as SEM-EDS or confocal microscopy, are needed to confirm the distribution of the active compound within the nanofibrous structure and provide definitive evidence of the encapsulation.

## 4 Conclusions

This study demonstrated that PBAT/PVP nanofiber mats produced by solution blow spinning (SBS) have adjustable structural and functional properties, depending on the composition of the mixture. The incorporation of PVP significantly impacted the fiber morphology, resulting in variations in diameter and uniformity associated with changes in the interactions between the polymer chains and solution properties. Biodegradation tests revealed comparable degradation behavior across the formulations, with minor variations in the cumulative evolution of CO_2_, suggesting that the PBAT/PVP ratio affects the extent of biodegradation rather than the degradation rate. Furthermore, herbicide-loaded systems exhibited inhibition of seed germination, suggesting the potential application of these biodegradable mats as carriers of agricultural input. However, the encapsulation and distribution of the active compound within the nanofiber structure could only be inferred indirectly and require direct structural characterization, load capacity, and release kinetics to confirm the efficiency of these systems for their intended use.
